# The Interior of a Fashionable House

**Published:** 1855-01

**Authors:** 


### THE INTERIOR OF A FASHIONABLE HOUSE.

The *New York Times*, in a recent article on the "Morals of Fashionable Society," gives the following picture of the interior of an elegant residence, together with a portraiture of its mistress.

Let us enter that magnificent house with the brown stone front, and the winter garden jutting out from the main building, and one of the shutters in each window half closed, in order that the passers by may see that they are of the finest satin wood, picked out with gold. Passing through large drawing rooms *en suite* and divided by Marisco arches, we will softly enter the little boudoir on the left, where in the midst of the dim light that steals through the windows, stained a pale rose color, a lady reclines in a luxurious fauteiul reading. She is very lovely. Her dress is orientally rich and picturesque, but an air of terrible languor overspreads her beauty. While she is finishing that bad chapter in the worst of Paul de Kock's novels, we will tell you a few facts about her.

She was brought up to make a good match. She left a fashionable boarding school at the age of fifteen, with a perfect knowledge of dancing, the French language, and the art of putting out a shawl. A summer at a fashionable wateringplace prepared her morals and her manners for a larger sphere of society, and at sixteen she made her *debut.* She was the rage for two years, and went everywhere, but when verging on her nineteenth year, her mother observed with alarm that her appearance was beginning to fade, and it was determined that she should marry forthwith; so she became Madam before she was twenty. And what sort of a heart did she bring her husband? One with youth and freshness, and purity to sanctify their intercourse? Pshaw! what has she to do with such things! She was never young. She was brought up from the cradle to look upon every thing as moral that was expedient, and when she married, married for an establishment. Her husband soon found out her heartlessness, and took to clubs when his business hours were over. And she has nothing to do all day long, but to sit in satin chairs, and read corrupt French novels, and flirt with idle young men. Over that luxurious home there floats no angel of happiness. Its owners lead a dreary, sensual life, miserable and splendid. None of those peaceful joys which less fashionable people know are ever to be found there. Virtuous love shuns the place, and its mistress presides there in her beauty and magnificence, haunted by a nameless agony, like those gorgeous monarchs in the hall of Eblis, who reigned in unceasing pain. And thus her life wears on till some day the bubble bursts.

And there was what might have been a happy home destroyed forever by a vicious system of education, and a false system of society. If that girl had been brought up to look upon marriage as a sacred responsibility, instead of an advantageous settlement—if her heart had not been indurated by her mother's ceaseless counsels to encourage only such men as would make a good *partie*—if she had been taught that woman had other duties in life to fulfil beside dancing well and managing a man—things might have been different.

Our fashionable society in this city is a sham, from beginning to end. It is utterly unsound, depraved, and unnatural—a deceptive piece of rotten wood, made to look shiny with French polish, and glittering with the phosphorescent light of corruption — a copper cent, trying its very best to look like a five franc piece, and what is worse, in nine cases out of ten, succeeding.

